# Longevity pathways and maintenance of the proteome: the role of autophagy and mitophagy during yeast ageing

**DOI:** 10.15698/mic2014.04.136

**Published:** 2014-04-07

**Authors:** Belém Sampaio-Marques, William C. Burhans, Paula Ludovico

**Affiliations:** 1 Life and Health Sciences Research Institute (ICVS), School of Health Sciences, University of Minho, Braga, Portugal.; 2 ICVS/3B’s - PT Government Associate Laboratory, Braga/Guimarães, Portugal.; 3 Dept. of Molecular and Cellular Biology, Roswell Park Cancer Institute, Buffalo, NY 14263, USA.

**Keywords:** nutrient-sensing pathways, autophagy, mitophagy, chronological lifespan, replicative lifespan, ageing, yeast

## Abstract

Ageing is a complex and multi-factorial process that results in the progressive accumulation of molecular alterations that disrupt different cellular functions. The budding yeast *Saccharomyces cerevisiae* is an important model organism that has significantly contributed to the identification of conserved molecular and cellular determinants of ageing. The nutrient-sensing pathways are well-recognized modulators of longevity from yeast to mammals, but their downstream effectors and outcomes on different features of ageing process are still poorly understood. A hypothesis that is attracting increased interest is that one of the major functions of these “longevity pathways” is to contribute to the maintenance of the proteome during ageing. In support of this hypothesis, evidence shows that TOR/Sch9 and Ras/PKA pathways are important regulators of autophagy that in turn are essential for healthy cellular ageing. It is also well known that mitochondria homeostasis and function regulate lifespan, but how mitochondrial dynamics, mitophagy and biogenesis are regulated during ageing remains to be elucidated. This review describes recent findings that shed light on how longevity pathways and metabolic status impact maintenance of the proteome in both yeast ageing paradigms. These findings demonstrate that yeast remain a powerful model system for elucidating these relationships and their influence on ageing regulation.

## INTRODUCTION

Ageing is a complex and multi-factorial biological process driven by genetic, environmental and stochastic factors that lead to cellular degeneration and the progressive decline of multiple physiological functions. The decline of these functions constitutes one of the most important risk factors for the development of numerous age-related diseases. The possible combinations of events occurring during ageing make it particularly challenging to reveal the interplay and hierarchical order of these events as well as to study their consequences at a molecular level. In the past few decades, various model organisms (invertebrate and vertebrate) and systems have been extensively exploited to investigate the mechanisms of ageing, including its determinants, risk factors and the so called "longevity pathways". The budding yeast *Saccharomyces cerevisiae *offers an attractive eukaryotic model that has dramatically accelerated ageing research 1,2,3,4. It has been particularly useful for elucidating cellular ageing mechanisms and for the identification of promising potential anti-ageing drugs such as rapamycin, resveratrol and spermidine that were first identified and characterized in yeast before finding common ground in the broad scientific community and confirmed in higher organisms.

*S. cerevisiae* provides two separate, but overlapping paradigms for ageing studies, replicative lifespan (RLS) and chronological lifespan (CLS). RLS measures the number of daughters cells produced by a single mother cell before senescence and constitutes a model for studying the ageing of mitotically active cells. CLS determines the time that cells survive in a non-dividing state after depletion of nutrient sources in stationary phase and allows for the study of ageing in post-mitotic cells.

In this review, the role of metabolism on primary damage responses and the relevance of mitochondria-specific dysfunction and reactive oxygen species as ageing determinants will be discussed in the context of novel insights into interventions that extend yeast lifespan, such as caloric restriction and hormesis. This review will particularly focus on the interconnection between nutrient-sensing pathways and the maintenance of proteostasis during ageing and its study in both yeast paradigms of ageing.

## THE CRITICAL ROLE OF NUTRIENT-SENSING PATHWAYS IN AGEING

Many of the interventions that extend lifespan of diverse organisms, including yeast, flies, worms, fish, rodents, and rhesus monkeys, decrease activity of nutrient-signaling pathways. In yeast, a reduction in the activity of two nutrient-sensing pathways, the target of rapamycin (TOR)/the serine-threonine kinase Sch9 5,6 and the Ras/protein kinase A (PKA) 7,8, can extend the two types of yeast lifespans. Deletion of *TOR1* or *SCH9* causes an increase in both CLS and RLS 5,6,9,10. Regulation of CLS by Sch9 can occur independently of Tor1, given that Sch9 can be specifically phosphorylated by the Pkh1/2 kinases, which activity is regulated by phytosphingosine (PHS), an intermediate in sphingolipid metabolism 11. A downregulation of sphingolipid synthesis results in yeast CLS extension indicating that Sch9 may function as an integration point of both nutrient- and sphingolipid-derived signals for appropriate regulation of yeast CLS 12,13. The contribution of Sch9 to the modulation of RLS can also occur independently of Tor1 through mechanisms involving the Sucrose Non-fermenting protein (Snf1) kinase, the yeast orthologue of the mammalian AMPK kinase, as discussed below 14.

Extension of CLS by reduced TOR activity depends on the transcription factors Gis1 and Msn2/4, which are activated by Rim15 and lead to an increase in many protective systems including glycogen accumulation, glycerol accumulation, anti-oxidant enzymes and mechanisms related to the maintenance of proteostasis, such as heat shock proteins (HSPs) and autophagy (reviewed in 1). Interestingly, RLS extension promoted by Tor1 inhibition is also dependent on the transcription factors Msn2/4 but this mechanism appears to be dependent on a sirtuin deacetylase, Sir2, as discussed further on 15.

The second pathway mediating yeast longevity extension is Ras/PKA. A reduced activity of PKA results in an increase in yeast lifespan in both ageing paradigms 7,16. Nevertheless, abrogation of *RAS1* and *RAS2* that lead to the activation of the PKA pathway, results in opposite effects on the replicative and chronological lifespans. Deletion of *RAS1 *gene increases RLS but is associated with a slight decrease of CLS, while deletion of *RAS2* decreases RLS, but intriguingly extends CLS 16,17,18. The activation of the transcription factors Msn2/4 is a crucial event for the extension of CLS 5, and probably of RLS 15, promoted by reduced Ras/PKA.

Overall, the results of many studies support the model that extension of yeast lifespan by the nutrient-sensing pathways requires the up-regulation of antioxidant enzymes, particularly Mn-dependent superoxide dismutase (Sod2), which scavenges the superoxide anions 19,20. This is also supported by the elevated levels of Sod2 detected in cells deleted in *SCH9* 21 or *RAS2 *16 and by the reduction of superoxide anion levels observed after inactivation of TOR pathway 21,22. Nevertheless, the pro-longevity effects attributed to the activation of a general stress response by decreasing the nutrient-sensing pathways activity seem to be also associated with an increase of mitochondria function. In fact, it has been established that lack of mitochondrial respiration severely affects the survival of stationary phase cells and thus the CLS 22,23,24,25. In addition, long-lived cells deleted of *TOR1* 22,24,25 or *SCH9 *26 display an increased respiratory capacity.

Inactivation of TOR/Sch9 and Ras/PKA nutrient-sensing pathways can be achieved by caloric restriction (CR), which is an experimental manipulation that extends the lifespan of a variety of eukaryotic organisms from yeast to mammals 10. CR in yeast corresponds to the reduction of glucose content in growth media from the regular 2 to 0.5% or, in some studies, 0.05%. Under these conditions, mitochondria respiration is released from glucose repression at an earlier time point and nuclear genes, important for mitochondrial biogenesis and function, are upregulated. Importantly, it was recently shown that extension of CLS by CR requires mitochondria respiration during exponential growth, which increases stress resistance, relieving the need for respiration in stationary phase 25. Under CR conditions, Rim15 kinase is also released from Tor1, Sch9 and Ras/PKA inhibition, and superoxide dismutases and other oxidative stress defenses are upregulated in a Rim15 partially dependent fashion 19. Recently, we have shown that CR also promotes pro-longevity effects in a Rim15-independent manner 20,27. We found that CR or inactivation of catalases also extends CLS by inducing elevated levels of hydrogen peroxide, which inhibit the accumulation of intracellular superoxide anions by activating superoxide dismutases by a Rim15-independent pathway 20,27. These findings point to a hormetic role for hydrogen peroxide during ageing. This study as well as other recent studies challenge prior paradigms for understanding the role of reactive oxygen species (ROS) in ageing and the free radical theory of ageing that posits oxidative damage to macromolecules as a primary determinant of lifespan 28. It has also been shown that in some scenarios, longevity is enhanced by inactivation of oxidative stress defenses or is correlated with increased ROS and oxidative damage 29. More recent findings established that increased mitochondrial ROS levels produced during cells growth reduce the accumulation of ROS at later stages of survival and consequently increased longevity 24. The early production of ROS was suggested to be dependent on the inhibition of TOR activity, contributing to the extension of CLS 24. Recently, the beneficial effects of hormesis were shown to also involve epigenetic alterations 30. It was demonstrated that yeast DNA damage response kinases, Tel1 and Rad53, homologs of the mammalian DNA damage response kinases ATM and Chk2, mediate a hormetic mitochondrial ROS longevity signal that extends yeast CLS 30. This pathway senses mitochondrial ROS in a manner independent and distinct from the nuclear DNA damage response, but connected with telomere functional status through the inactivation of a histone demethylase 30. Nevertheless, it is suggested that although epigenetic effects are essential for the adaptive response elicited by ROS, the transcriptional changes mediated by Msn2/4 and Gis1 are also a fundamental part of the hormetic plan that mediates CLS extension.

The results of numerous studies suggest that the determinants of yeast replicative and chronological lifespans are distinct, but overlap 3,31. The fact that both replicative and chronological lifespans are extended in response to CR and other interventions decreasing nutrient-sensing pathways demonstrated that both yeast ageing paradigms share conserved features with the ageing processes in evolutionarily divergent multicellular organisms 3. Nevertheless, it remains unclear whether similar downstream molecular events are common to both yeast ageing paradigms. Both yeast ageing paradigms are interconnected; it was recently reported, for example, that chronologically aged yeast cells show a proportional reduction in RLS 32,33,34. Recently, it was also reported that CR protects chronologically aged cells from a reduction in RLS, suggesting that the metabolic state and mitochondrial function of stationary phase cells determines their replicative potential upon transfer to growth conditions 31.

Although the evidence from many studies implies that both yeast ageing paradigms invoke conserved determinants of ageing in multicellular eukaryotes 1,3, many issues remain to be explored. These include elucidation of the different relationships between the nutrient-sensing pathways and the so called metabolic linkers such as Snf1 and sirtuins, mitochondria homeostasis and their impact in proteome homeostasis of aged cells.

## METABOLIC SENSORS: AMPK AND SIRTUINS

The role of nutrient-sensing pathways as determinants of longevity has been extensively explored, but the relevance to ageing of new nutrient hubs, often called metabolic sensors, is now attracting attention and has become the focus of intense investigation. One metabolic sensor is Snf1, which functions as an energy sensor that is able to reprogram cellular metabolism in order to restore normal energy levels essential to sustain cell metabolism and to the cellular response to different stresses 14,35,36. Activation of Snf1 is dependent on the cellular ratio AMP/ATP but not on AMP allosteric regulation. Snf1 is also regulated by PKA activity, upon low glucose stress, independently of the AMP/ATP ratio 37. In contrast to mammalian AMPK, Snf1 is negatively regulated by Tor1 38. Snf1 kinase activity can also be independently controlled by acetylation of Sip2, a regulatory subunit of the Snf1 complex 14. In addition, Sch9, the yeast homolog of Akt and S6K, was shown to be a common downstream target of Snf1 complex and of Tor1 14. Notably, Snf1 activity, required for transcription of glucose repressed genes, increases in yeast aged cells, even when glucose is abundant 39,40, and this has detrimental effects on lifespan 41. Sip2 acetylation is suggested to enhance physical interaction with Snf1 and thereby antagonize its catalytic activity 14. This could explain why Sip2 is able to suppress the negative effects of Snf1 on RLS extension and why abrogation of *SIP2* decreases lifespan 42. The detrimental effects of Snf1 in RLS can also be explained by its activation of Sch9, independently of the TOR pathway, which might lead to loss of proteostasis. Snf1 was also shown to be a modulator of CLS 43,44. Deletion of *SNF1* results in shortening of CLS 44, a phenotype that was hypothesized to be related to the role of Snf1 in promoting respiration and autophagy 45,46. These findings suggest that in contrast to its detrimental effect in RLS, Snf1 could be necessary for fitness during CLS, which is a phenotype resembling the promoting effects of AMPK activation under CR in metazoans 47,48,49.

Sirtuins, a highly conserved group of NAD+ dependent protein deacetylases that responds to high NAD+/NADH ratios, are another type of metabolic linker. Sirtuins are considered to be ‘‘master regulators’’ of eukaryotic ageing due to pioneering work showing that deletion of *SIR2* decreases yeast RLS, whereas its overexpression increases RLS 50. It was proposed that CR extends RLS by activating Sir2 deacetylase activity, either through an increase in the intracellular NAD+/NADH ratio 51, and/or a reduction in the nicotinamide concentration 52. Nicotinamide, a by-product of the deacetylation reaction, is a potent noncompetitive Sir2 inhibitor but CR elevates the expression of Pnc1, a nicotinamidase, thereby promoting Sir2p deacetylase activity (reviewed in 52,53). Although other deacetylases have been implicated in CR-mediated extension of RLS by suppressing rDNA recombination, the role of these sirtuins in mediating CR effects on yeast RLS remains controversial 54. Nevertheless, although Sir2 mammalian orthologues were shown to be linked to increased longevity, the mechanism by which Sir2 acts to extend yeast RLS does not seem to be relevant to ageing in multicellular eukaryotes 2.

**Figure 1:**
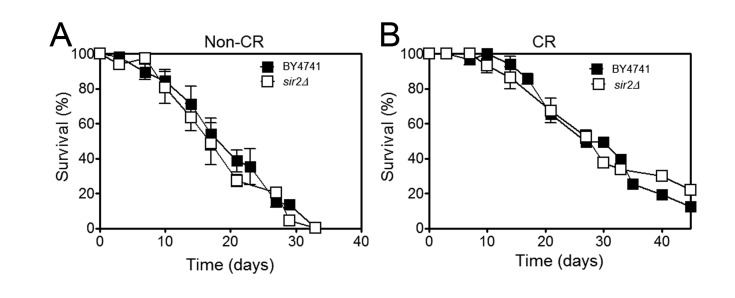
FIGURE 1: *SIR2* deletion does not have a major impact on CLS either in normal growth conditions or under caloric restriction. Chronological lifespan of wild type (background BY4741) or *sir2*Δ cells submitted to **(A)** non-caloric restriction (non-CR) or **(B)** caloric restriction (CR) conditions. All experiments were performed in synthetic complete (SC) medium containing yeast nitrogen base and glucose, as a carbon source, supplemented with the appropriate amino acids and bases. The concentration of glucose used was 2% in non-CR conditions or 0.5% to promote CR. Cells were incubated at 26°C with shaking at 150 rpm. Cultures reached stationary phase 2 days later and this was considered day 0 of CLS. Survival was assessed by counting colony-forming units (CFUs) beginning at day 0 of CLS (when viability was considered to be 100%), and then again every 2-3 days until less than 0.1% of the cells in the culture were viable. The data represent mean ± SEM of three biological independent replicas. No statistical significance was obtained between the CLS curves presented both in panel A and in panel B, as determined by two-way ANOVA.

The role of Sir2 in yeast CLS appears to be different and it has been mainly assigned as having a pro-ageing role in CLS. Depending on the strain background and growth media, deletion of *SIR2* either has no effect or induces a moderate increase of CLS (reviewed in 53). Abrogation of *SIR2* combined with CR and/or mutations in the yeast *SCH9* or *RAS1/2* causes a dramatic CLS extension 44,55. Overexpression of *SIR2* has no effect on CLS but reduces the CLS of cells lacking Sch9 activity 55. Nevertheless, the effects of Sir2 in CR-mediated CLS longevity are also controversial. Although it was shown that Sir2 antagonizes CLS extension promoted by CR 55, it was later shown that CR extends CLS of *S. cerevisiae *independently of the sirtuins including Sir2 54. Consistent with these observations, our data showed that deletion of *SIR2* does not have a major impact on CLS either in normal growth conditions or under CR (Figure 1). This suggests that the longevity promoting effects of CR are independent of Sir2 although it remains unclear whether CR has an impact on Sir2 activity. The fact that deletion of *SIR2* in combination with reduced PKA or Sch9 activity (promoted by CR) leads to an increase of the expression of several stress-resistance genes and a decrease on the rate of DNA mutations that accumulate with age in post-mitotic conditions could explain the absence of CLS effects under CR conditions.

Although Sir2 is not associated with CR-promoting CLS extension, treatments with resveratrol, an ageing modulator that mimics CR, result in CLS extension by mechanisms that depend on Sir2 56. This finding supports the hypothesis that under CR conditions, Sir2 can have a function in regulating lifespan, but by as yet undiscovered mechanisms. Our recent studies have shown that Sir2 upregulates macroautophagy and mitophagy during CLS 57. In fact, during CLS under conditions of proteotoxic stress induced by the heterologous expression of the human alpha-synuclein, a protein associated with Parkinsons' disease, Sir2 transcriptionally regulates *ATG8* and *ATG32* 57. These findings show that similar to its mammalian orthologue, SIRT1, Sir2 is also a macroautophagic regulator. Interestingly, resveratrol, a polyphenolic compound found in red wine that is known to prolong lifespan in lower eukaryotes via sirtuin activation 56, regulates autophagy in a mechanism that depends on Sir2 but is independent of TOR 58. Remarkably, although independent of Sir2, it was shown that the deacetylation of histone H3 in response to spermidine is associated with increased autophagy, reduced oxidative stress and CLS extension 59. These results indicate that the role of Sir2 on CLS is complex and raise the interesting possibility that Sir2 is part of an elaborate signaling network that regulates autophagy and ageing.

## PROTEOSTASIS COLLAPSE, AUTOPHAGY AND AGEING

Ageing and some ageing-related diseases are linked to impaired protein homeostasis or proteostasis 60,61. As mentioned above, long-lived phenotypes are usually associated with increased stress resistance and altered metabolism, particularly mitochondria bioenergetics. A hypothesis that is becoming well accepted is that one of the major function of these "longevity pathways" is to contribute to the maintenance of the proteome during ageing 62. To maintain proteostasis, cells possess quality control mechanisms such as the degradation of proteins by the proteasome or the lysosome/vacuole that function in a coordinated fashion 63,64,65. Different studies have demonstrated that proteostasis is altered with ageing 64 and that the inefficient removal of non-functional molecules and cellular components associated with a general decline in the cellular housekeeping mechanisms seem to have a pivotal role in the progression of ageing 66.

**Figure 2:**
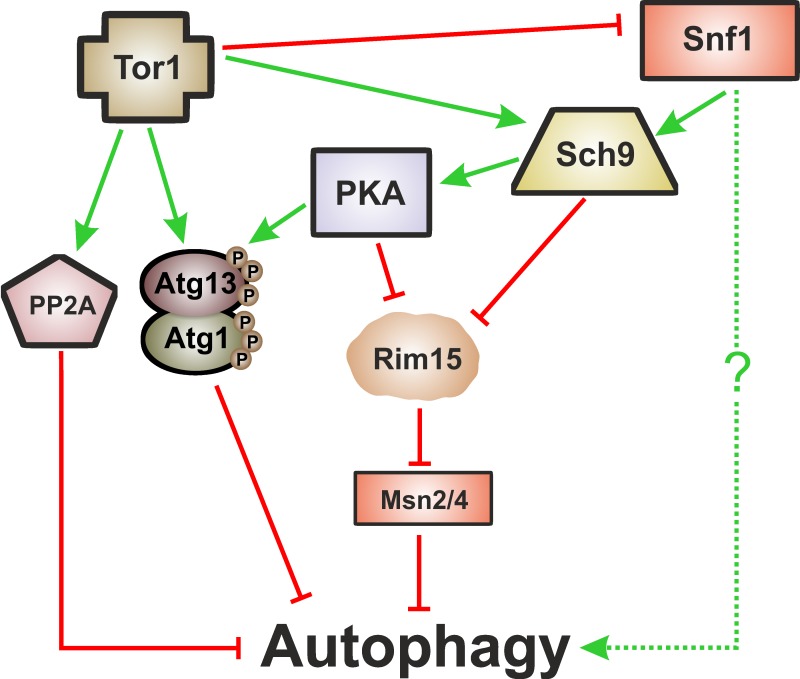
FIGURE 2: Autophagy regulation by nutrient-sensing pathways. Tor1 is the main negative regulator of autophagy. Tor1 can directly exert its negative regulation through phosphorylation of Atg13 and Atg1 or through its downstream target, PP2A (serine/threonine protein phosphatase 2A), a negative regulator of autophagy. Atg1 and Atg13 could also be phosphorylated by PKA, at residues distinct from those targeted by Tor1. PKA and Sch9 pathways are also negative regulators of autophagy. Apparently, TOR and PKA pathways operate in parallel, whereas Sch9 acts in parallel with PKA and partially dependent on TOR. Regulation of autophagy by Sch9 and PKA can be mediated by the inhibition of Rim15 and the Msn2/4 transcription factors. Tor1 is also able to inhibit the activity of the metabolic sensor Snf1, which is a positive regulator of autophagy by still unknown mechanisms. Green arrows indicate interactions that induce autophagy, red bars indicate inhibition. See the text for details.

Macroautophagy, herein called autophagy, is one of the cellular proteolytic systems that guarantees the quality of proteins and organelles via their sequestration within double-membrane vesicles called autophagosomes that are delivered to lysosomes/vacuoles for degradation 67. Importantly, and as described above, autophagy is a common downstream target of the so-called "longevity pathways", which points to a crucial cytoprotective role of autophagy during ageing. These signaling pathways are negative regulators of autophagy with partial overlapping branches and as yet undetermined hierarchical connections 66. Tor1 is considered to be the main negative regulator of autophagy 68,69 either through its direct phosphorylation of Atg proteins such as Atg13 or through a signaling cascade involving the phosphorylation of Tap42, which activates the catalytic subunits of PP2A (the serine/threonine protein phosphatase 2A), a negative regulator of autophagy 70. Atg1 and Atg13 are also targets of PKA, which negatively regulates autophagy through their phosphorylation at residues distinct from those targeted by Tor1 71,72. TOR and PKA pathways appear to operate in parallel and to be involved in an elaborated network regulating autophagy (reviewed in 73). Like Tor1 and PKA, inactivation of Sch9 also induces autophagy 72,74. Sch9 acts in parallel with PKA and its activity is partly dependent of TOR 75. Regulation of autophagy by Sch9 appears to be different from the post-translational mechanisms implied by Tor1 and PKA above described and it is partly mediated by the inhibition of Rim15 (a positive regulator of autophagy) and the Msn2/Msn4 transcription factors 75. PKA dependent regulation of autophagy is also partially dependent on Rim15 (Figure 2) 74.

Mutant cells defective in autophagy-related genes exhibit reduced CLS in SD minimal medium compared to control strains 76. This phenotype could be suppressed by increasing the availability of essential and non-essential branched side chain amino acids by a mechanism that likely involves inactivation of Gcn4, which regulates general amino acid control 76.

Autophagy has been shown to be required for the extension of yeast CLS promoted by CR 44,76,77 but not for CR-mediated yeast RLS extension 78. These and other reports indicate that the interconnection between autophagy, CR, and longevity in yeast is still unclear. While autophagy seems dispensable for CLS extension during CR in a low glucose synthetic complete media 44, some genes involved in vacuolar membrane homeostasis and indirectly implicated in autophagy are required for lifespan extension during CR promoted by transferring cell to water 79,80. A recent study showed that autophagy is upregulated by the two CR interventions mentioned above but while autophagy seems to be always required for full extension of CLS during caloric restriction promoted by water wash, its requirement for CR longevity effects produced by low glucose is dependent on strain background 77. In both cases autophagy seems to contribute to the maintenance of respiration proficiency during ageing 77.

The picture of autophagy’s function in CLS and particularly in CR-mediated lifespan extension is even hazier if we consider the intervention of the metabolic linkers Snf1 and Sir2 on autophagy regulation and ageing. For instance, in the absence of nitrogen, the depletion of glucose is a positive signal for autophagy induction but in this situation, the Snf1 kinase is involved in regulation 45, although the details have not yet been elucidated (Figure 2).

Curiously, anti-ageing drugs such as resveratrol, spermidine and rapamycin are autophagic regulators/activators 81. While rapamycin stimulates autophagy in a TOR-dependent fashion, resveratrol and spermidine elicit a TOR-independent autophagy by distinct pathways. Sirtuins are required for resveratrol induced autophagy 82 but not for spermidine-stimulated autophagy 59,81.

Thus, a great deal of evidence supports the model that autophagy and ageing are coordinately regulated by a network of different signaling pathways, with partial overlapping branches.

## MITOCHONDRIAL DYNAMICS, MITOPHAGY AND BIOGENESIS DURING AGEING

Dysfunctional mitochondria can generate ROS, release cell death-inducing factors, such as cytochrome *c*, into the cytosol, or generally burden the metabolic machinery of the cell by decreasing the efﬁciency of ATP generation 83. Therefore, mitochondria need to be constantly repaired or degraded to prevent additional damage to the cell. Consequently, maintaining mitochondria homeostasis is extremely important during ageing 84, which is accompanied by a decline in mitochondrial turnover caused by reduced mitochondrial biogenesis and/or mitochondrial degradation 85. Mitophagy is a selective form of autophagy in which severely impaired mitochondria are degraded 86. The regulation of the mitophagy process is still poorly understood. Genomic screening for yeast mutants defective in mitophagy using a library of non-essential deletion strains identified genes involved in diverse pathways, such as membrane trafficking, protein modification/degradation, lipid metabolism or mitochondrial metabolism 87. Mechanistically, Atg32 was found to be essential to mitophagy and has been proposed to function as a mitochondrial receptor that during mitophagy interacts with Atg11, an adaptor protein for selective types of autophagy 88,89.

Mitophagy is intimately connected to mitochondrial dynamics and thus to the mitochondria fission/fusion machinery, which in turn is inactivated when a bioenergetic collapse takes place. Although in mammalian cells mitophagy is impaired when mitochondrial fission is blocked, in yeast the role of mitochondrial dynamics in mitophagy and mitochondrial quality control has been proposed to be independent of mitochondrial fission machinery (see for discussion 90). Mitophagy regulation in yeast also seems to occur by other mechanisms distinct from those operating in higher eukaryotic cells. This is demonstrated by the fact that drugs affecting the electron transport chain are not strong inducers of mitophagy in yeast, as they are in mammalian cells. Such differences may reflect the fact that yeast have evolved to prefer fermentation to respiration, and unlike some mammalian cells, they can dilute out damaged or superfluous organelles by division.

Whi2, a protein required for full activation of the general stress responses, was shown to act as a mitophagy-promoting factor 91. Whi2 was shown to interact with Msn2 92 and to have an important regulatory role of Ras/PKA pathway 93. *WHI2* deletion results in the hyperactivation of PKA and a dramatic decrease in Msn2/4 activity. Thereby, Whi2 has been proposed to be a key player in adapting the complex network of signaling pathways in response to the nutritional status of the cell 90,93. Nevertheless, the role of Whi2 in yeast ageing is still unclear although it was reported that *WHI2* mutant cells have a reduction of RLS 94.

**Figure 3:**
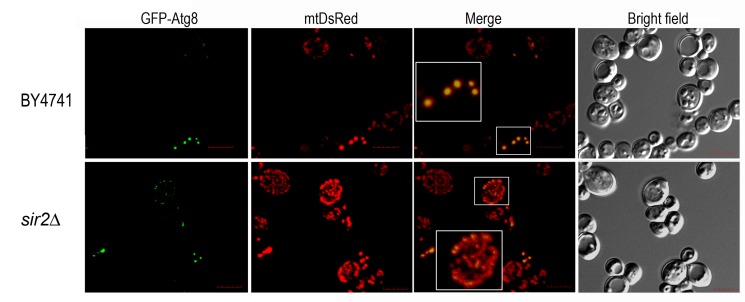
FIGURE 3: *SIR2* deleted cells exhibit increased mitochondrial mass and altered mitochondria network. Wild type and *sir2*Δ (background BY4741) cells expressing, GFP-Atg8 and mtDsRed were analyzed for mitophagy by confocal fluorescence microscopy. The cells were collected and analyzed at day 3 of chronological lifespan. Mitophagy was analyzed by the pattern of co-localization between GFP-Atg8 and mtDsRed, as exemplified. Images were acquired in a confocal Olympus FLUOVIEW microscope with an Olympus PLAPON 60X/oil objective, with a numerical aperture of 1.35. GFP and DsRed were excited with and argon laser and a helium-neon laser (GFP: 488 nm excitation; DsRed: 559 nm excitation). Background reduction was performed with appropriate saturation levels using software FV1000 (Olympus) and Adobe Photoshop CS. Image stacks for analysis were acquired with sequential steps of 0.25 to 0.5 µm per plane in the z-direction and a total thickness of 4-6 µm. The acquired stacks were rendered with FV1000 software. Scale bars: 5 µm.

An important pathway in mitochondrial homeostasis is the retrograde signaling response. This pathway compensates the accumulation of mitochondrial dysfunctions that occur during ageing and crosstalks with other relevant signaling pathways including pathways involved in metabolic stress response such as TOR pathway (reviewed in 95). Mitophagy occurring in post logarithmic phase is controlled by Aup1, a phosphatase that localizes to the mitochondrial intermembrane space, which regulates Atg33 96. Aup1 also mediates the dephosphorylation and nuclear import of Rtg3, a key component of the retrograde signaling pathway, which is also required for post-log phase mitophagy 97. Although it is well accepted that mitophagy has an important role in the maintenance of mitochondria homeostasis during ageing, this is still an overlooked aspect of ageing, and relatively little is known about the proteins involved in regulating selective autophagy. It is suggested that mitophagy, probably activated by the retrograde response, is responsible for extending RLS in mtDNA-deficient strains 98. As far as CLS is concerned, both starvation-dependent and stationary phase, it is known that mitophagy is partially regulated by two separate mitogen-activated protein kinase (MAPK) pathways. Bck1, a MAPK kinase, was identified in a screen for mitophagy-defective strains 87 and was shown that together with upstream and downstream kinases and the cell surface sensor Wsc1 is required for mitophagy 99. The second MAPK, Hog1, appears to be also regulated by a cell surface sensor, Sln1 99. The downstream targets of these MAPKs are still not identified but is expected the involvement of certain transcription factors. However, regulation of mitophagy during CLS is not restricted to MAPKS control. Our previous studies have shown that in CLS measurements made under conditions of proteotoxic stress, Sir2 is an important regulator of the transcription of *ATG32* encoding a mitochondrial protein that confers selectivity during mitophagy 57. Microscopic analysis of mitophagy revealed that *SIR2* mutant cells have an increased mitochondrial mass and suggested alterations in mitochondria networks, although the CLS of *SIR2* mutant cells is not significantly different from wild-type cells (Figure 3 and 1). These results also reinforce the role of Sir2 in the regulation of mitophagy during CLS and point to an exploitation of *SIR2* mutant cells to unveil more about mitochondria homeostasis during ageing. Nevertheless, under proteotoxic stress, we have shown that a deregulated increase in mitophagy leads to a shorter CLS, indicating that similar to autophagy the selective degradation of mitochondria has to be maintained around a threshold above which it aggravates ageing and reduces CLS 57. This seems to be counter-intuitive as autophagy/mitophagy are viewed as protective mechanisms. However, it’s clear that a tight equilibrium between mitochondrial dynamics, mitophagy and biogenesis must be maintained during ageing.

## CONCLUSION

Accumulating evidence points to a role for conserved nutrient-sensing pathways in the regulation of ageing dependent and independent of general stress responses. Both yeast ageing paradigms have contributed enormously to the understanding of how inactivation of conserved nutrient-sensing pathways impact on longevity. Although some nutrient-sensing pathways, downstream effectors and outcomes are distinct in the two yeast lifespan models, replicative and chronological, important shared features have also been detected. Nevertheless, it appears that the CLS model shares more aspects of the ageing process in higher eukaryotic cells than does RLS. What also appears to be the case is that together with increased stress responses and mitochondria bioenergetic capacity, the reduced activity of nutrient-sensing pathways, observed in long-lived phenotypes, is also a regulating process crucial for cellular proteostasis. Nevertheless, many questions remain unanswered, such as the role of and interconnections between nutrient-sensing pathways and "metabolic sensors" such as Snf1 and sirtuins, in the coordinated regulation of ageing. In the past, the role of mitochondria degradation (mitophagy)/biogenesis during ageing has been largely ignored. Although it is recognized that the maintenance of functional mitochondria and the degradation of dysfunctional ones is a fundamentally important component of ageing, the regulation of these events and how they impact lifespan is poorly understood. This is even more important in the context of ageing, during which biogenesis, and particularly mitochondria biogenesis, is quite limited and cells have to simultaneously integrate catabolic and anabolic signals to avoid triggering cellular pathways that will ultimately culminate in death. Therefore, one of the main challenges in ageing research is to understand how the events that constitute hallmarks of cellular ageing are regulated and interconnected. However, our ability to make sense of the complexity of the networks and their branches is limited. In the last decades, yeast has become not only the leading model for eukaryotic cell biology but also the pioneer organism that has facilitated the establishment of an entirely new approach to study biological modules (cells, pathways, networks, regulation) called systems biology. Systems biology will be helpful to understand ageing and lifespan determinants through for example large-scale analyses of gene expression and transcriptional networks, genetic interactions, protein-protein interactions, proteomes, phosphoproteomes, acetylomes, metabolomes or fluxomes. Therefore, we believe that yeast still have important secrets to divulge relevant to these questions and that yeast studies are likely to produce many additional novel findings relevant to ageing and ageing interventions in mammals, including humans.
